# Synergistic Ice Inhibition Effect Enhances Rapid Freezing Cryopreservation with Low Concentration of Cryoprotectants

**DOI:** 10.1002/advs.202003387

**Published:** 2021-01-29

**Authors:** Tie Chang, Oyawale Adetunji Moses, Conghui Tian, Hai Wang, Li Song, Gang Zhao

**Affiliations:** ^1^ Department of Electronic Science and Technology University of Science and Technology of China No. 96 Road Jinzhai Hefei Anhui 230027 China; ^2^ National Synchrotron Radiation Laboratory CAS Center for Excellence in Nanoscience University of Science and Technology of China Hefei Anhui 230029 China; ^3^ CAS Key Laboratory for Biomedical Effects of Nanomaterials and Nanosafety CAS Center for Excellence in Nanoscience National Center for Nanoscience and Technology Beijing 100190 China; ^4^ University of Chinese Academy of Sciences Beijing 100049 China; ^5^ School of Biomedical Engineering Anhui Medical University Hefei Anhui 230032 China

**Keywords:** cryopreservation, ice inhibition, synergistic effect, tungsten diselenide‐polyvinyl pyrrolidone nanoparticles

## Abstract

Despite recent advances in controlling ice formation and growth, it remains a challenge to design anti‐icing materials in various fields from atmospheric to biological cryopreservation. Herein, tungsten diselenide (WSe_2_)‐polyvinyl pyrrolidone (PVP) nanoparticles (NPs) are synthesized through one‐step solvothermal route. The WSe_2_‐PVP NPs show synergetic ice regulation ability both in the freezing and thawing processes. Molecularly speaking, PVP containing amides group can form hydrogen bonds with water molecules. At a macro level, the WSe_2_‐PVP NPs show adsorption‐inhibition and photothermal conversation effects to synergistically restrict ice growth. Meanwhile, WSe_2_‐PVP NPs are for the first time used for the cryopreservation of human umbilical vein endothelial cell (HUVEC)‐laden constructs based on rapid freezing with low concentrations of cryoprotectants (CPAs), the experimental results indicate that a minimal concentration (0.5 mg mL^−1^) of WSe_2_‐PVP NPs can increase the viabilities of HUVECs in the constructs post cryopreservation (from 55.8% to 83.4%) and the cryopreserved constructs can also keep good condition *in vivo* within 7 days. Therefore, this work provides a novel strategy to synergistically suppress the formation and growth of the ice crystalsfor the cryopreservation of cells, tissues, or organs.

## Graphical abstract

Tungsten diselenide (WSe_2_)‐polyvinyl pyrrolidone (PVP) nanoparticles (NPs) are synthesized through one‐step solvothermal route. The WSe_2_‐PVP NPs show synergetic ice inhibition based on the hydrogen bonds, adsorption‐inhibition, and photothermal conversation effects. Therefore, the WSe_2_‐PVP NPs significantly facilitate the high‐efficiency cryopreservation of cell constructs using rapid freezing method with low concentration of cryoprotectants.

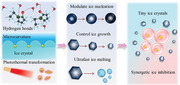

## 1 Introduction

Water is a fundamental element for all life on earth and will turn into ice when the temperature is below the freezing point. However, ice formation on materials surface causes serious threat to the activities of human beings, ranging from technological applications to economic fields.^[^ ^1^, ^2^, ^3^, ^4^, ^5^, ^6^ ^]^ For instance, the supercool water accumulating on the surface of aircraft, marine power system, industrial power, and energy units (such as, transmission lines, power network towers, or photovoltaic power plants) not only decreases the operating efficiency, but also leads to great threats to human life.^[^ ^7^, ^8^, ^9^ ^]^ Therefore, understanding and regulating the formation and growth of ice crystals are of significant importance in various fields such as biological science, atmosphere science, ocean environment, and aerospace.^[^ ^10^ ^]^ Fortunately, nature has endowed some nature organisms (such as some fishes, insects, plants in the polar region) with a unique way to weaken and avoid ice damages for cold adaptation.^[^ ^11^ ^]^ Antifreeze proteins (AFPs) produced in their body play an important role in ice regulation due to selectively adsorption to the surface of ice crystals.^[^ ^12^, ^13^ ^]^ Generally, the AFPs possess the special functions in controlling ice crystals in following three aspects: 1)depression of freezing point leading to a thermal hysteresis (TH), 2) ice recrystallization inhibition (IRI), and 3) dynamic ice shaping.^[^ ^14^, ^15^ ^]^ These unique ice tuning properties that nature endowed the AFPs with, afford us a novel method to suppress the formation and growth of ice crystal, which is of great significance in the fundamental research for the cell/tissue cryopreservation, geology, climate science, as well as, the design of anti‐icing materials.^[^ ^16^, ^17^, ^18^ ^]^ However, the rarity, complexity, and low thermal stability of AFPs restrict their widespread use in practical applications.^[^ ^19^ ^]^ In order to create artificial ice inhibitors with similar ice regulation ability of AFPs, a lot of efforts have been devoted to mimic the function of AFP using macromolecular polymer and nanomaterials. For example, Gibson and co‐workers used polyvinyl alcohol (PVA) as addictive for cryopreservation and found the only 0.1% PVA polymer can inhibit ice growth during thawing and improved cell recovery post cryopreservation.^[^ ^14^, ^20^ ^]^ Similarly, Wang et al. discovered that graphene oxide (GO) can adsorb onto ice crystal surface and retard the growth of ice due to the formation of hydrogen bonds between GO and ice‐like water.^[^ ^21^ ^]^ Similarly, oxide quasi‐carbon nitride quantum dots (OQCNs) show ice shaping ability and TH activity,^[^ ^22^ ^]^ and molecular dynamics simulation analysis demonstrated that the in‐plan structure of OQCNs can match with ice crystal lattice. Besides, proline, poly(ampholyte), and some molecule aryl‐glycosides also show ice inhibition ability and were applied in cell cryopreservation to reduce ice cryoinjury.^[^ ^23^, ^24^, ^25^ ^]^ Nevertheless, the ice inhibition materials reported above only possess single ice suppression ability such as modulating ice formation or restricting ice growth.

The process of deicing is as important as tuning ice crystals in the cooling stage. The capability of removing ice can be greatly enhanced by heating effect. To this end, realizing rapid melting ice by utilizing photothermal or magnetothermal conversion nanoparticles (NPs) (e.g., GO nanosheet, gold nanoparticles, Fe_3_O_4_, etc.) has been reported.^[^ ^26^, ^27^, ^28^, ^29^, ^30^, ^31^, ^32^, ^33^, ^34^ ^]^ Among these methods, laser is particularly attracted owing to its high energy density, high efficiency, and easy operation.^[^ ^35^ ^]^ For instance, significant progresses of laser‐induced photothermal therapy have been made in treating tumors, drug delivery, and catalysis, or others.^[^ ^36^, ^37^, ^38^ ^]^ The major challenges faced in current cryopreservation mainly include the ice injury both in the freezing and thawing stages, the toxicity of cryoprotectant (CPA), and the permeable damages et al.^[^ ^39^ ^]^ Among these damage mechanisms, the ice formation, growth, and recrystallization are critical problems and will cause fatal mechanical damage to cells.^[^ ^40^ ^]^ Hence, achieving uniform and fast rewarming is essential for cell/tissue cryopreservation. In order to generate more uniform and faster rewarming rates, compared with previously reported nanoparticles such as gold and GO nanomaterials, the transition metal dichalcogenides (TMDCs) family have great potential in the field of biomedical engineering because of their tunable local surface plasmonic resonance, high optical transmittance, high thermal conductivity, ultrahigh carrier mobility, and larger surface area.^[^ ^37^, ^41^ ^]^ Among the TMDC family, tungsten diselenide (WSe_2_) based nanomaterial with excellent photothermal transformation performance has never been explored for cell cryopreservation. Moreover, the constituent elements of WSe_2_ (tungsten and selenium) are important for human health, albeit in low concentrations, and the biocompatibility of WSe_2_ can be significantly improved by surface modification, thus ensuring biosafety to biological samples.

The polyvinyl pyrrolidone (PVP), as a kind of typical addictive applied in cell cryopreservation, can effectively reduce cryoinjuries. Furthermore, it has been reported that the PVP can form hydrogen bonds with water/ice molecules,^[^ ^42^ ^]^ indicating that its ice regulation ability plays an important role in decreasing cryodamage during cryopreservation. In this study, to our knowledge, we first integrate passive and active ice regulation ability, that is, ice formation and growth regulation, as well as rapid melting ice property by adopting bottom‐up synthesis approach, which necessitated the synthesis of metallic (1T) phase WSe_2_ coated with PVP. More importantly, this manifests economically viable and reliable facile fabrication platform to synthesize WSe_2_‐PVP with an ideal surface morphology and suitable chemical composition that can facilitate a large‐scale production. As schematically illustrated in

**Figure 1:**
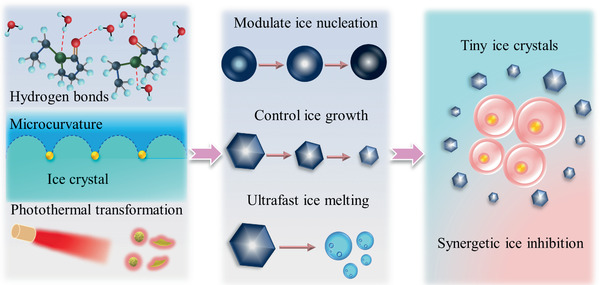
Schematic illustrations of synergetic ice inhibition mechanisms of WSe_2_‐PVP NPs and facilitate the cell cryopreservation efficiency.

**Figure** **1**, due to the synergistic effects through the formation of hydrogen bonds, adsorption‐inhibition effect, and excellent photothermal transformation performance, the WSe_2_‐PVP NPs both show ice regulation and ultrafast ice melting performances. Based on the synergetic ice modulation effect, the WSe_2_‐PVP NPs remarkably reduce the ice injury in cryopreservation and greatly enhance survival efficiency of cells. Collectively, the novel and multifunctional WSe_2_‐PVP NPs open a new window in controlling ice crystals and ultrarapid melting ice, having great potentials in various applications including biomedical research, cryopreservation, transportation, and anti‐icing materials.

## 2 Results and Discussion

### 2.1 Synthesis and Characterization of Tungsten Diselenide‐Polyvinyl Pyrrolidone Nanoparticles

WSe_2_‐PVP NPs were synthesized through a one‐step solvothermal reaction, and the schematic illustration of synthesis is illustrated in Figure S1, Supporting Information. First, selenourea crystals (0.5 mmol) and tungsten hexachloride powders (WCl_6_) (0.1 mmol) were mixed with PVP (0.1 g or 0.5 g) in 20 mL *N*,*N*‐dimethylformamide. Then, the mixture was kept in autoclave and heated at 220 °C for 24 h under sealed condition. Finally, the samples were cooled to room temperature, washed several times with alcohol and dried in oven at 60 °C for 24 h. Owing to the chelating‐coordinating effect between the lone‐pair electrons of PVP carbonyl oxygen and the unoccupied 5d orbitals of W,^[^ ^41^ ^]^ the PVP molecular chains are able to graft onto the surface of WSe_2_ and guide the growth of nanosheets in the process of hydrothermal. In this work, the ratios comparing WSe_2_ and PVP (1–0, 1–1, 1–5, 1–10, and 1–15) were based on the quantity of the reactants utilized, which was entirely dependent on molecular weight of individual precursors. For instance, the ratio 1–1of WSe_2_‐PVP was fabricated with both precursors of equal mass (0.1 g) while that of ratio 1–5 possesses PVP weight in the factor of five times those of WSe_2_ reagents (WCl_6_‐0.1 mmol per 0.04 g and selenourea‐0.5 mmol per 0.06 g). Interestingly, the WSe_2_‐PVP nanosheets could combine to form nanoflower shape by increasing the concentration of PVP to 1.0 and 1.5g. Therefore, the morphological difference is dictated by the proportion of precursor PVP polymer in each sample. As reported, increase in the percentage ratio of PVP:WSe_2_ precursors initiates a phenomenon called “orientated attachment” which makes the nanosheets to spontaneously pile on each other.^[^ ^43^ ^]^ Herein, respective types 1–1and 1–5 WSe_2_‐PVP NPs are nanosheets structure corresponding to the addition of 0.1, and 0.5 g PVP while the types 1–10 and 1–15 WSe_2_‐PVP NPs are nanoflowers structure representing the addition of 1 and 1.5 g PVP.

To investigate the morphology of the prepared WSe_2_‐PVP nanosheets, the scanning electron microscope (SEM) and transmission electron microscope (TEM) images are shown in Figure S2a, Supporting Information, which demonstrates that the samples display uniform nanosheet structure. The high‐magnification HRTEM image signifies that the nanosheets are composed of 3–8 layers, and the distance of every interlayer is 1.6 nm, implying ultrathin WSe_2_‐PVP nanosheets. The inserted figure further proved the good dispersion of the WSe_2_‐PVP NPs. Similarly, the nanoflowers structure of WSe_2_‐PVP NPs is also confirmed with SEM images as shown in Figure S2b, Supporting Information. Nevertheless, such differing morphological layout does not alter the chemical structure and composition of WSe_2_‐PVP NPs, thus the nanosheet was chosen as characterization object. Figure S2c, Supporting Information, demonstrates that carbon, nitrogen, and oxygen (C, N, and O) are major elemental composition of our as‐prepared nanosheets (WSe_2_‐PVP NPs). To further investigate the structure and composition of WSe_2_‐PVP NPs, X‐ray powder diffraction (XRD), Fourier transform infrared (FT‐IR) spectra, and Raman spectra analyses were used and the results are exhibited in Figure S2d–f, Supporting Information. The XRD results correspond to standard WSe_2_ and the strong peak in Raman spectra is 240 cm^−1^ further demonstrating the peak results are consistent with previous study on WSe_2_.^[^ ^44^ ^]^ It is worth noting that the 002 peak position in XRD shifts toward lower Braggs angle (Figure S2d, Supporting Information), indicating the PVP intercalated into WSe_2_ layers,^[^ ^45^ ^]^ which is also proved by the peak of 1286 and 1653 cm^−1^in FT‐IR spectra.^[^ ^41^, ^46^ ^]^ To characterize the chemical states of the as‐prepared WSe_2_‐PVP, the X‐ray photoelectron spectroscopy (XPS) measurement results are illustrated in Figure S2g–i, Supporting Information, in which the C 1s, W 4f, Se 3d were detected (Figure S3, Supporting Information), respectively.^[^ ^44^, ^47^ ^]^ The peaks at 284.6, 285.8, 287.4 eV demonstrate the C—C, C—N, C—N = O functional groups.^[^ ^48^ ^]^ All in all, on basis of chelating‐coordinating effect between the lone‐pair electrons of PVP carbonyl oxygen and the unoccupied 5d orbitals of W, as demonstrated in Figure S4, Supporting Information, implying successfully synthesized WSe_2_‐PVP NPs.

### 2.2 Ice Nucleation Modulation during Cooling Process

**Figure 2:**
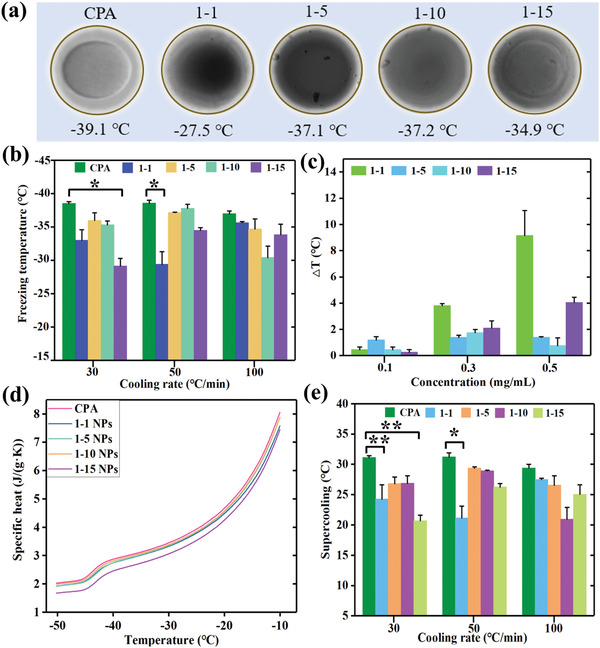
Ice nucleation regulation performance assessment of WSe_2_‐PVP NPs during cooling process. a) Typical optical ice crystal image comparison of CPA solution with and without WSe_2_‐PVP NPs. b) Ice nucleation temperature comparison of CPA solution with and without WSe_2_‐PVP NPs under different cooling rates. c) The difference value of ice nucleation temperature between pure CPA solution and with WSe_2_‐PVP NPs under different concentrations. d) The comparison of specific heat of CPA solution with and without WSe_2_‐PVP NPs. e) The degree of supercooling of CPA solution with and without WSe_2_‐PVP NPs. * indicates a statically significant difference relative to control (pure CPA without WSe_2_‐PVP NPs). *p < 0.05, **p < 0.01.

Modulating ice nucleation formation is significant to reduce cryodamage to cryopreserved samples during cooling process.^[^ ^6^ ^]^ Especially, the low ice nucleation temperature can result in higher supercooling temperature, and thus generating more pointed dendritic shape of ice crystals, which will lead to fatal injury to cells in cooling process. Therefore, the ability of tuning ice nucleation temperature and decreasing supercooling is vital to promote the survial efficiency of cell cryopreservation. In order to explore the influence of WSe_2_‐PVP NPs on the CPAs solution during cooling process, we analyzed the temperature of ice nucleation formation under different conditions. Due to the randomness and instantaneity of ice nucleation formation, it is extremely difficult to measure accurate ice nucleation temperature. Hence, here the temperature at which ice crystals began to appear in the view was roughly regarded as ice nucleation temperature. As shown in **Figure** **2**a, the nucleation temperature of CPA solution is about −39.1 °C, whereas, the nucleation temperature is about −27.5, −37.1, −37.2, and −34.9 °C for the solution with four types of WSe_2_‐PVP NPs, respectively. It indicated that the addition of WSe_2_‐PVP NPs is able to regulate ice nucleation temperature. In term of the theory of heterogenous nucleation,^[^ ^6^ ^]^ the WSe_2_‐PVP NPs play the role of nucleating matrix and thereby promoting the formation of crystals nucleus. Afterward, the ice nucleation behavior under different cooling rates is further assessed. Generally, the addition of WSe_2_‐PVP NPs can increase nucleation temperature and promote the ice nucleation formation, but the ice regulation ability of different types of WSe_2_‐PVP NPs is variable under different cooling rates as demonstrated in Figure 2b. The possible reasons of sensitivity of different types of WSe_2_‐PVP NPs are caused by the content of PVP distributed onto WSe_2_ surface and the shape of nanoparticles. Moreover, the ice nucleation temperature with different concentrations of WSe_2_‐PVP NPs are also studied. It is discovered that the degree of ice modulation temperature △*T* (the difference value of ice nucleation temperature between CPA solution and with WSe_2_‐PVP NPs) is the highest when the concentration of 1–1 WSe_2_‐PVP NPs is 0.5 mg mL^−1^, roughly increasing up to 10 °C compared with pure CPA solution (Figure 2c). According to the mechanism of thermodynamics, the addition of WSe_2_‐PVP NPs decreased the specific heat compared with pure CPA and strengthened the heat transformation capacity of this solution (Figure 2d). As depicted in Figure 2e, the supercooling temperature of different solution under three cooling rates was measured. Overall, the supercooling temperature can be decreased when the WSe_2_‐PVP NPs were added in the CPA solution. Based on the theory of heterogenous nucleation,^[^ ^49^, ^50^ ^]^ the addition of WSe_2_‐PVP NPs decreased the nucleation potential barrier, and thus reducing the supercooling temperature. Meanwhile, different types of WSe_2_‐PVP NPs show different regulation capacity for supercooling, which is probably closely related with the size and shape of different WSe_2_‐PVP NPs. As previously reported by Wang et al.,^[^ ^6^ ^]^ the ice nucleation temperature can be affected by the size of GO nanosheet. Here it can be hypothesized that the size and the shape of WSe_2_‐PVP NPs have closed relationship with the ice nucleation temperature of CPA solution. Molecularly, the amide functional group of PVP also forms hydrogen bonds with water, thus alternating the fraction of ice‐like water molecules and breaking the arrangement of ice‐like water molecules, which can affect the ice nucleation temperature.^[^ ^51^ ^]^ However, the exact ice modulation mechanisms of WSe_2_‐PVP NPs that affects the dynamic and structure of ice molecules need to be further studied.

### 2.3 Ice Recrystallization Inhibition during Thawing Process

**Figure 3:**
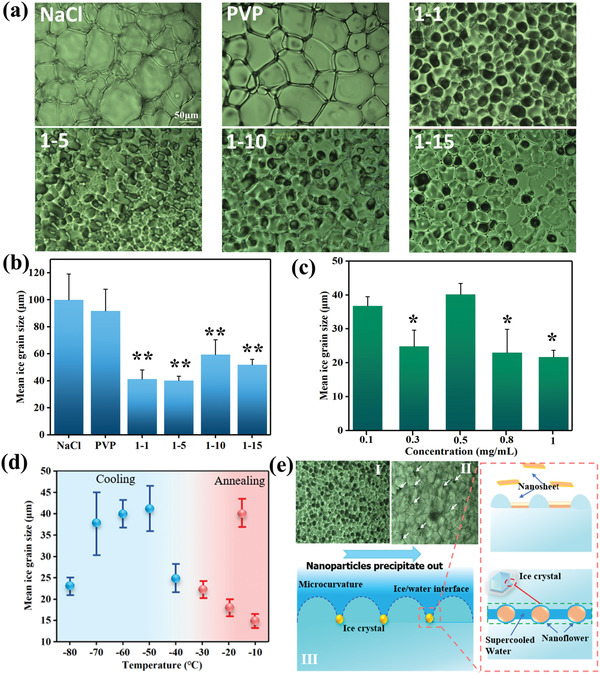
IRI activity of WSe_2_‐PVP NPs. a) Microscopic images of ice crystals grown in the NaCl solution (0.9%) with and without WSe_2_‐PVP NPs. b) The different IRI ability of various WSe_2_‐PVP NPs samples. c) Effects of concentration of WSe_2_‐PVP NPs on IRI activity. d) Influences of quenching temperature and annealing temperature on IRI ability of 0.5 mg mL^−1^ WSe_2_‐PVP NPs. e) The possible adsorption‐inhibition mechanism of WSe2‐PVP NPs on the ice crystal surface. I),II): After WSe2‐PVP NPs precipitated out from solution, the sizes of ice crystals gradually increased, which proved the IRI effect of WSe2‐PVP NPs due to adsorption inhibition. III): Schematic illustrations show the nanoflower and nanosheets are confined between ice grains and further induce the micro‐curvatures on ice crystal surface. * indicates a statically significant difference relative to control group with pure NaCl solution. *p < 0.05, **p < 0.01.

In respect to IRI, previous researchers have reported that the pure PVP has slight influence on suppressing ice recrystallization during annealing process.^[^ ^52^ ^]^ Here, the ability of tuning ice growth for WSe_2_‐PVP NPs is evaluated by the experimental process of “splat cooling” (Figure S5, Supporting Information)^[^ ^21^, ^53^ ^]^ which has been widely applied to study IRI phenomenon. To assess the ice growth inhibition capability, WSe_2_ nanosheet that intercalated by different amount of PVP molecules was studied. As shown in **Figure** **3**a, the ice grain size of 0.9% NaCl solution after adding PVP (0.5 mg mL^−1^) and WSe_2_‐PVP NPs (0.5 mg mL^−1^) was observed, and the optical image analysis of ice grain size indicates the obvious IRI capability of WSe_2_‐PVP NPs. Generally, different kinds of WSe_2_‐PVP NPs show different IRI influences in 0.9% NaCl solution. Compared with pure NaCl solution and PVP solution, the smallest ice grain size can be observed in the WSe_2_‐PVP of 1–5 nanosheets solution which is much smaller than pure NaCl solution and the solution after adding pure PVP polymer with the same concentrations (0.5 mg mL^−1^) as depicted in Figure 3b. Interestingly, although the nanoflowers surface possesses more PVP molecules, the inhibition effect of ice crystals is not as good as nanosheets, suggesting the geometrical shape and size of NPs can also affect the growth of ice crystal. Figure 3c reveals that the mean ice grain size decreases from 36.8 to 21.7 µm as the concentrations of 1–5 WSe_2_‐PVP nanosheets increase from 0.1 to 1 mg mL^−1^ (w/v), suggesting that the capability of controlling ice growth and IRI effect can be enhanced by increasing the nanosheets concentration. Thus, the above results indicate that the WSe_2_‐PVP NPs have the capability to restrict the ice crystals growth and recrystallization in term of the quantitative comparison of ice grain size.

Next, the influence of quenching and annealing temperature on inhibiting ice crystals growth capacity of 1–5 WSe_2_‐PVP nanosheets are further investigated. As shown in Figure 3d, the quenching temperature has observable effects on the IRI activity of WSe_2_‐PVP NPs. According to the experimental results analyses, the mean ice grain sizes do not directly depend on quenching and annealing temperatures. When the quenching temperature is −50 °C, the mean ice grain size is biggest, this may be caused by the higher probability of heterogeneous ice nucleation at this temperature.^[^ ^51^, ^54^ ^]^ Ice crystals possess the largest area at the annealing temperature of −15 °C, which probably is caused by the homogeneous diffusion and growth of quasi‐liquid layer on ice surface (Figure S6, Supporting Information),^[^ ^55^ ^]^ thus resulting in larger ice grain size at −15 °C annealing temperature.^[^ ^56^, ^57^ ^]^ Meanwhile, the stable mean area of ice crystals also displays excellent IRI robustness of WSe_2_‐PVP NPs after undergoing four quenching and annealing cycles (Figure S7, Supporting Information), demonstrating the great potential of WSe_2_‐PVP NPs in the application of IRI.

As shown in Figure 1, the probable mechanisms of inhibiting ice formation and growth are ascribed to the molecular level, chemical structure, and macroscopic adsorption‐inhibition effect of WSe_2_‐PVP NPs. It can be hypothesized that the reasons and mechanisms of ice inhibition of WSe_2_‐PVP NPs should be caused by the following three points: (i) Molecularly, the N and O atoms existing in PVP are likely to form hydrogen bonds with H_2_O due to the bigger electronegativity of N and O atoms, serving as hydrogen‐binding sites, and thus endowing the PVP polymer with the capability of regulating ice formation and growth.^[^ ^22^ ^]^ As shown in Figure S8, Supporting Information, the differential scanning calorimetry (DSC) results also demonstrate that the melting enthalpy of CPA solution gradually decreases with the increase of PVP, indicating that the fraction of non‐freezing water in this solution raises due to the molecular interaction between PVP and water. Also, it can be hypothesized that due to the formation of hydrogen bonds between PVP coated onto surface of WSe_2_ and ice molecules, and further influencing adsorption of WSe_2_ on ice interface. Consequently, the WSe_2_‐PVP NPs show IRI effect to some extent. It is worth noting that the distance between N atoms and oxygen atoms on the primary prism plane of the ice lattice plays a significant role in controlling ice crystal.^[^ ^58^, ^59^ ^]^ The formed hydrogen bonds between PVP and H_2_O further hinder the ordered ice lattice, which can be verified by the analysis of molecular dynamic simulation.^[^ ^21^, ^60^, ^61^ ^]^ (ii) The honeycomb chemical structure arrangement of WSe_2_ can match with the hexagonally shaped ice crystals, and possess similar function in GO.^[^ ^62^ ^]^ Also, the GO and OQCNs can preferentially adsorb on ice crystals, which is described as Langmuir‐type kinetic model.^[^ ^21^, ^22^ ^]^ (iii) Furthermore, the shape and size of NPs has different influence on the IR effects. According to the experimental results (Figure 3a–c), the WSe_2_‐PVP NPs preferably adsorb onto surface of ice crystals and lead to micro curvatures (Kelvin effect), and this effect is also proved by the results of separating out WSe_2_‐PVP NPs from solution through the comparison of ice sizes as demonstrated in Figure 3e(I,II). As previously reported about the ice inhibition mechanism of AFPs,^[^ ^4^, ^63^, ^64^ ^]^ initially, the AFPs can bind to the basal or prism faces of ice crystals. As more and more ice layers produce small convex surfaces, and then the curvature of newly grown ice increases, ultimately the size of ice crystals is at a limited value, therebyrestricting ice growth due to Kelvin effect. Additionally, the nanoflowers with more PVP molecules do not have more observable IRI effect, which is probably due to the sizes of some nanoflowers, larger than the distance between ice and supercooled water interface, thus weakening the property of mimic AFPs in IR process as schematically illustrated in Figure 3e(III). Hence, the rationalized geometrical shape of NPs has important influence on adsorption‐inhibition effects, also suggesting that dynamic regulation between ice/water interface and generating curvatures of ice layers enhances antifreeze activity. Therefore, the outstanding capability of controlling ice crystals endows the WSe_2_‐PVP NPs with great potential in the applications of anti‐icing materials.^[^ ^65^, ^66^, ^67^ ^]^

### 2.4 Photothermal Conversion Property for Recrystallization/Devitrification Inhibition

**Figure 4:**
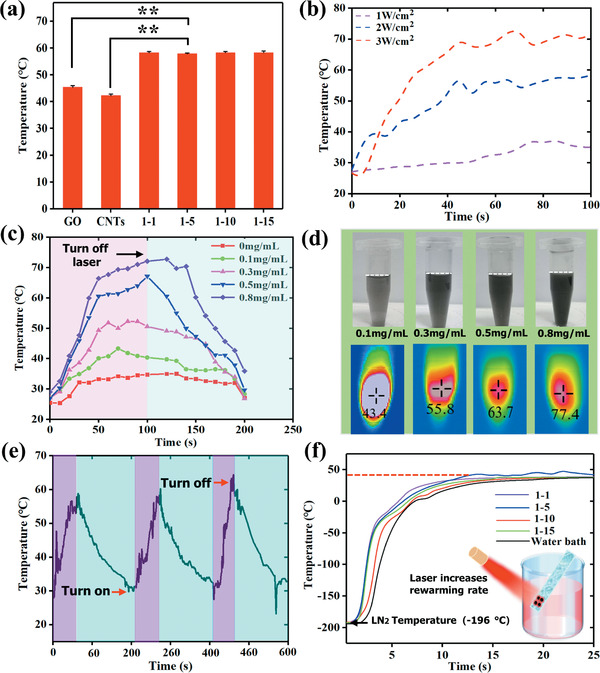
The photothermal transformation effect of WSe_2_‐PVP NPs. a) The photothermal transformation property comparison of four types of WSe_2_‐PVP NPs with GO and CNTs under same conditions. * indicates a statically significant difference relative to control group. **p < 0.01. b) Temperature changing curves of 1–5 WSe_2_‐PVP NPs under 1, 2, 3 W cm^−2^ laser power. c) The influence of concentrations of WSe_2_‐PVP NPs on photothermal transformation effect. d) Infrared thermography images of WSe_2_‐PVP NPs with various concentrations. e) The temperature change curve for 0.5 mg mL^−1^ WSe_2_‐PVP NPs with laser power of 3 W cm^−2^ for 30 s and then turn off for 170 s with three cycles. f) Temperature change curves of vitrification solution with four types of WSe_2_‐PVP NPs under laser irradiation from nitrogen to water bath. The insert image indicates that the photothermal transformation effect accelerates thawing rate and inhibits devitrification and/or recrystallization.

Ultrarapid rewarming rate is essential in the fields of fundamental research including anti‐ice materials, food, precious source transportation, and tissue/organ cryopreservation.^[^ ^33^, ^35^, ^68^ ^]^ Recently, for the vitreous cryopreservation of cells, we have discovered that the photothermal conversion effect of GO can be utilized to achieve rapid thawing and thereby avoiding cell damages caused by devitrification and/or recrystallization.^[^ ^28^ ^]^ Hereby, we propose that the novel WSe_2_‐PVP NPs can be utilized to modulate ice growth and achieve rapid rewarming (from −196 to 37 °C). **Figure** **4**a displays the photothermal transformation effects of four types of 0.5 mg mL^−1^ WSe_2_‐PVP NPs compared with same concentration of GO and CNTs under 808 nm laser irradiation. It can be seen that the WSe_2_‐PVP NPs exhibit superior photothermal transformation performance than that of GO and CNTs. We further measured the photothermal conversion effects of 1–5 type WSe_2_‐PVP nanosheets (0.5 mg mL^−1^) during 100 s under laser irradiation of various powers. As shown in Figure 4b the temperature profile of nanosheets solution exhibit substantial increase under laser irradiation and positively correlated with its power intensity. When the laser power is 3 W cm^−2^, the temperature of the nanosheets solution can be increased to ≈70 °C, which is ≈40 °C higher than 1 W cm^−2^. In addition, the photothermal transformation effects are also correlated with the concentration of the nanosheets (Figure 4c), which is further confirmed with the infrared images as revealed in Figure 4d.

Moreover, to further measure the photothermal transformation robustness of the synthesized WSe_2_‐PVP NPs, 3 W cm^−2^ laser power was utilized to irradiate 0.5 mg mL^−1^ WSe_2_‐PVP NPs for 30 s and then cooled for 170 s to initial temperature. Figure 4e shows that temperature of nanosheets solution can increase to similar degree during three cycles of laser irradiation, indicating the photothermal capability of WSe_2_‐PVP nanosheets are stable during the laser irradiation. To further study the rewarming capability of WSe_2_‐PVP NPs in cryopreservation process, the temperatures of vitrified solution with four types of 0.5 mg mL^−1^ WSe_2_‐PVP NPs were monitored from plunging into liquid nitrogen (−196 °C) to water bath (37 °C) as illustrated in Figure 4f. The results indicate the photothermal thawing increases the rewarming rates compared with conventional water bath (Figure S9, Supporting Information), especially in the range of −196 to 0 °C that is in the risk temperature zone, which shows great potential in the field of cryopreservation. For cell cryopreservation, the dangerous temperature zone extending from −160 to 0 °C can generate fatal injury for thawed cells,^[^ ^39^ ^]^ thus rapid thawing rate is critical for cells to quickly pass through this temperature range and avoid ice cryodamages. The heating effect induced by WSe_2_‐PVP NPs enables the rewarming process to extensively accelerate, which implies that the photothermal transformation effect of WSe_2_‐PVP NPs promotes the rapid thawing and thus shortening the occurrence duration of recrystallization and/or devitrification.

### 2.5 Application of Tungsten Diselenide‐Polyvinyl Pyrrolidone Nanoparticles for Cryopreservation of Cell‐Laden Constructs

**Figure 5:**
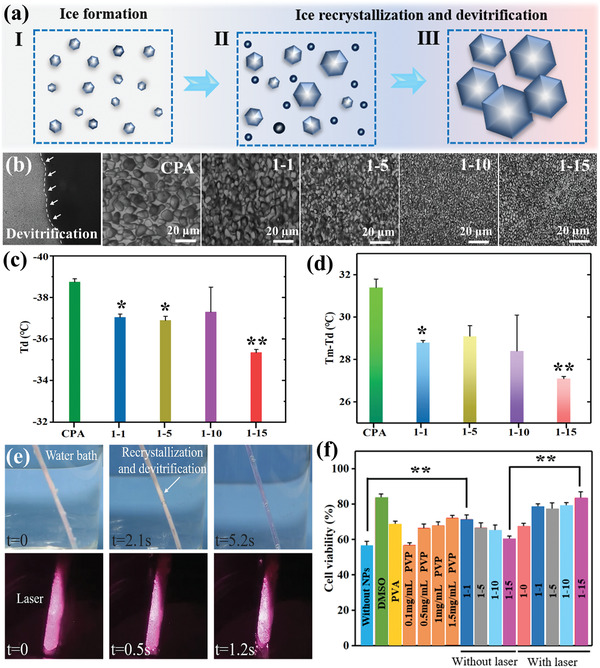
Synergetic ice inhibition effect of WSe_2_‐PVP NPs in the process of cryopreservation to enhance cell recovery. a) The schematic illustration of recrystallization and/or devitrification and IRI during warming process. b) The typical images of devitrification and ice recrystallization of CPA solution with and without WSe_2_‐PVP NPs in the thawing process. c) The occurrence temperature of devitrification of CPA solution with and without WSe_2_‐PVP NPs under 50 °C min^−1^ cooling rate and 20 °C min^−1^ warming rate. d) The comparison of temperature interval between devitrification and melting point of pure CPA solution and with WSe_2_‐PVP NPs. e) Devitrification and/or ice recrystallization duration with and without laser irradiation. f) Viability of HUVEC‐cell constructs post cryopreservation with different additives. * indicates a statically significant difference relative to control group. *p < 0.05, **p < 0.01.

Currently, the main cryopreservation methods can be divided into slow freezing and vitrification approaches.^[^ ^69^ ^]^ For the slow freezing strategy, it not only need complex cryogenic devices but also the ice injury limits the cryopreservation efficiency. Regarding vitrification strategy, the high concentration (e.g., 6–8 m) of penetrating CPAs such as dimethylsulfoxide (DMSO) will cause toxic and osmotic injury to cryopreserved cells.^[^ ^70^, ^71^ ^]^ To address the contradiction between ice formation and the toxicity of high‐CPA, different from conventional vitrification cryopreservation methods with high concentration of CPAs, our work proposed rapid‐freezing cryopreservation strategy using low concentration of CPAs (2 m) with the addition of WSe_2_‐PVP NPs. Although the CPA with or without WSe_2_‐PVP NPs can realize partial vitrification (Figure S10, Supporting Information) during cooling process, the low concentration of CPAs will induce the occurrence of metastable state of the solution and thus the ice recrystallization and/or devitrification are major restricted problems. As schematically viewed in **Figure** **5**a, as the ice crystals gradually grew, new ice nucleus occurred, and finally the large ice crystals cause cryoinjury to the cryopreserved biological samplesduring thawing process. The DSC analysis results demonstrated that the vitrification and devitrification will generate for the CPA solution (Figure S11, Supporting Information). Meanwhile, the results of cryomicroscope images further proved the occurrence of devitrification and recrystallization of CPA solution during warming process (Figure 5b). Additionally, after the addition of WSe_2_‐PVP NPs, the size of ice grain for the CPA solution is reduced, which is also consistent with the previous results of Figure 3. It should be noted that it is difficult to distinguish the recrystallization and devitrification during cell cryopreservation due to the randomness and instantaneity of ice nucleation formation.^[^ ^6^ ^]^

To show the application potential of WSe_2_‐PVP NPs in rapid‐freezing cryopreservation, we further quantitatively evaluate and analyze its ice regulation capacity for cell cryopreservation. When the CPAs solution was added into four types of WSe_2_‐PVP NPs, the extent of supercooling can be decreased owing to the strengthened heat transfer capacity (Figure 2d,e), and thus reducing cryodamage to cryogenic samples in cooling process. Especially, the hydrogel of cell‐laden construct can separate the cryopreserved cells and WSe_2_‐PVP NPs. The TEM image showed that few WSe_2_‐PVP NPs were observed in human umbilical vein endothelial cells (HUVECs) after incubation with HUVEC‐laden constructs and WSe_2_‐PVP NPs for 6 h at 4 °C (Figure S12, Supporting Information), which avoided the generation of cell internalization and interacting with cell membrane of nanoparticles. Also, it indicated that the WSe_2_‐PVP NPs can be easily removed post cryopreservation. In the thawing procedure, the WSe_2_‐PVP NPs delayed the occurrence of devitrification and/or recrystallization as shown in Figure 5c. Additionally, the fatal cryoinjury for cell cryopreservation is the risky temperature zone, that is the temperature gap (*T*_m_–*T*_d_) between recrystallization and/or devitrification (*T*_d_) and melting point (*T*_m_) during thawing procedure, because ice formation and large ice crystals will occur in this temperature gap. As demonstrated in Figure 5d, the WSe_2_‐PVP NPs significantly shorten the risky temperature gap due to the enhancement of heat transfer effect, suggesting that the ice injury can be decreased in this warming process. Furthermore, the excellent photothermal transformation property of WSe_2_ NPs also accelerates the rewarming rates for CPA solution and thereby greatly shortening the staying duration time between risky temperature zone (from −15 to −160 °C). As viewed in Figure 5e, compared with conventional water bath rewarming, the PS containing 2 m CPAs shows reduced ice crystals growth and shortened ice recrystallization duration time with the aid of laser. Hence, the unique and synergetic ice regulation ability can effectively reduce ice injury and endow the WSe_2_‐PVP NPs with great potential to promote rapid‐freezing cryopreservation.

For the cryopreservation experiment, the HUVEC‐laden constructs fabricated by using centrifugal microfluidics method were selected as cryopreserved samples (Figure S13a, Supporting Information). For the cryopreservation of cell‐laden constructs, how to overcome ice injury remains challenging, which will destroy the integrity of constructs and have negative effects on their further applications *in vivo*.^[^ ^72^ ^]^ Based on this, the WSe_2_‐PVP NPs that show synergetic ice modulation performance was used as addition for HUVEC‐laden constructs using rapid‐freezing method with low concentration CPAs. The toxicity evaluation was conducted to prove the biocompatibility of WSe_2_‐PVP NPs for HUVEC cells (Figure S14, Supporting Information). The viability data of HUVEC‐laden constructs post‐vitrification were displayed in Figure 5f. In sharp contrast, the survival rate increased from 55.8% (pure CPAs without WSe_2_‐PVP NPs) to 83.4% (CPA solution with addition of 0.5 mg mL^−1^ WSe_2_‐PVP NPs), which indicates that the additive of WSe_2_‐PVP NPs significantly enhances cryopreservation effect. Compared with the conventional cryopreserved method using toxic organic CPA (e.g., 10% v/v DMSO), the survival rate of cryopreserved cells with WSe_2_‐PVP NPs is comparable to that of DMSO (10% v/v). It is also discovered that the viabilities of cells post cryopreservation with the addition of PVP using same content with four types of WSe_2_‐PVP NPs, PVA (the typical ice inhibitor as shown in Figure S15, Supporting Information),^[^ ^76^ ^]^ pure WSe_2_ with laser irradiation, and WSe_2_‐PVP NPs without laser irradiation are lower than those using WSe_2_‐PVP NPs with laser thawing, further proving the synergetic ice modulation effect that contains the inhibition for the growth of small ice crystals and rapid thawing of WSe_2_‐PVP NPs can decrease the cryoinjury to cells. Hence, the proposed innovative synergetic ice tuning strategy that combines physical field (photo, electricity, and magnetism) responsive nanomaterials with ice inhibition polymers corporately improves cryopreservation efficiency, which will open a new avenue to design personalized and multifunctional nano‐cryoprotectants for rapid‐freezing cryopreservation of cells or organs using low concentration of CPAs.

**Figure 6:**
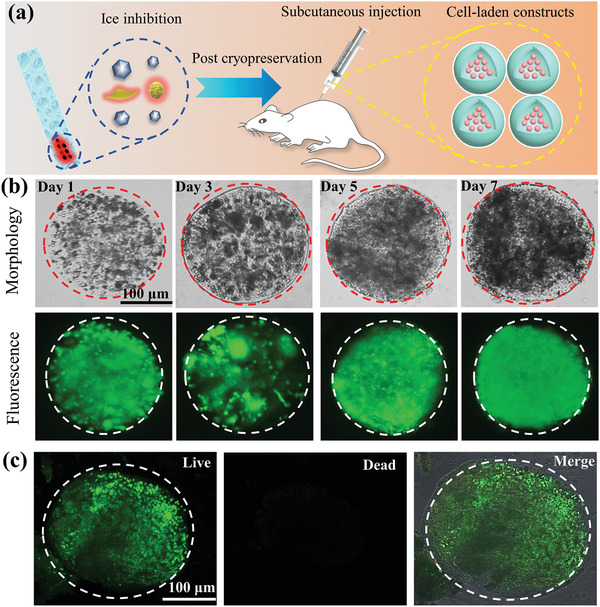
Viability evaluation of cryopreserved HUVEC‐laden constructs in vivo for different days. a) Schematic diagram of cryopreserved HUVEC‐laden constructs injection into the subcutaneous tissue of mouse back. b) Optical and fluorescence images of HUVEC‐laden constructs cultured in vivo for 1, 3, 5, 7 days, respectively. c) Confocal images of HUVEC‐laden constructs that have cultured at seventh day in vivo.

Cell‐laden constructs have great potential in tissue engineering and regenerative medicine, thus the efficient and reliable cryopreservation for cell‐laden construct is of great significance in future medical research and clinical treatment.^[^ ^64^, ^65^ ^]^ Currently, the assessment of cryopreservation efficiency is mainly studied in vitro but the survival situation of cryopreserved cells in vivo is not fully investigated. Therefore, in order to further evaluate the viabilities of cell‐laden constructs after cryopreservation and the application potential for regenerative medicine, the HUVEC‐laden constructs that underwent cryopreservation using WSe_2_‐PVP NPs were injected into hypodermis of mouse back as shown in **Figure** **6**a, and then the HUVEC‐laden constructs were cultured in vivo of mouse for 1, 3, 5, 7 days, respectively. The optical images display the integrity of cryopreserved cell‐laden constructs, as well as the structure of microcapsules remains intact. Meanwhile, the fluorescent images of cultured cell‐laden constructs show high viability within 7 days as demonstrated in Figure 6b, also indicating the WSe_2_‐PVP NPs can be utilized as an effective and biocompatible additive to improve cryopreservation efficiency. Moreover, the confocal microscopy was used to check the viabilities of cell‐laden constructs at the seventh day. As shown in Figure 6c, strong green fluorescence can be observed in the cell‐laden constructs suggesting most of the cells are in good state. Notably, the phenomena of cells aggregation demonstrate that the HUVEC‐laden constructs post‐cryopreservation using WSe_2_‐PVP NPs can be cultured in vivo and keep normal cell growth and proliferation. All in all, our innovative design of WSe_2_‐PVP NPs and its first application in cryopreservation provide a new strategy for regenerative medical research.

## 3 Conclusions

In summary, to address the cryodamage issues induced by the ice formation, growth, and recrystallization, we proposed a multiple ice regulation strategy by combining PVP polymer with 2D nanomaterial that possesses photothermal transformation effect. During cooling process, the WSe_2_‐PVP NPs can decrease the degree of supercooling of CPA solution and thereby reducing cryodamage to cryopreserved cells. Meanwhile, the uniformly dispersed WSe_2_‐PVP NPs can adsorb on the ice/water interface to restrict ice growth, and act as heating source and achieve global rapid rewarming to inhibit ice recrystallization and/or devitrification based on photo‐thermal conversion effect. Moreover, WSe_2_‐PVP NPs were first demonstrated as an effective ice inhibition agent for the cryopreservation of HUVEC‐laden constructs based on rapid‐freezing cryopreservation with low concentration of CPAs, and the cryopreserved construct samples maintained good viability in vivo within 7 days, which opens a new application direction for 2D materials in controlling ice and application in regenerative medical research. This work not only provides a novel strategy to simultaneously regulate ice formation and growth but also will further facilitate the developments of cell and organ cryopreservation, vaccine transportation, regenerative medicine, and tissue engineering. In the later study, we will design and synthesize multifunction‐integrated ice nano inhibitors such as the multiple photothermal or magnetocaloric 2D NPs that aremodified with other anti‐icing polymers (such as PVA, polyelectrolyte, proline et al.) and apply them in the fundamental research of controlling ice formation and its growth for the cryopreservation of more types of cells, tissues or organs.

## 4 Experimental Section

##### Materials

Selenourea (Reagent grade −99.97%) and tungsten hexachloride (WCl_6_) were obtained from Alfa Aesar. PVP was available from Sinopharm Chemical Reagent Co., Ltd. N and was used to fabricate different types of WSe_2_‐PVP NPs. PVA (The viscosities of PVA‐1, PVA‐2, and PVA‐3 are 2.8, 3.3, and 5.0 mpa.s, respectively) and other commercial cryoprotectant such as DMSO, glycerol, hetastarch (HES), biological sugars were purchased from Aladdin Ltd.

##### Characterization of Tungsten Diselenide‐Polyvinyl Pyrrolidone Nanoparticles

The surface morphology was analyzed via a TEM (H‐700, Hitachi, Ltd., Tokyo, Japan). Meanwhile, energy dispersive spectrometer was performed to measure surface elements of WSe_2_‐PVP NPs. Chemical state analysis of samples was characterized using XRD (X'Pert Pro, Netherlands). The chemical bonds and functional groups of synthetic WSe_2_‐PVP NPs were characterized using FT‐IR spectral (Nicolet 8700, Thermo Nicolet, Madison, USA) and Raman spectra (Jobin‐Yvon, Horiba Group, France). The XPS measurement equipment was conducted to analyze the chemical composition of the synthetic WSe_2_‐PVP NPs.

##### Ice Nucleation and Inhibition Experiment

To assess the ice inhibition activity, cryomicroscopy study was carried out using a Linkam FDCS196 cryostage with a digital camera. For the ice nucleation experiment, dispersive ultrasmall water droplet was placed at the center of solid substrate and put onto the cryostage to be tested. The whole experiment process was conducted in biosafety environment to avoid possible contaminant. IRI evaluation was performed via the “splat cooling method” as reported in previous reference.^[^ ^21^ ^]^ Briefly, a 20 µL of dispersion solution was dropped onto the surface of cooled silicon (−60 °C) from 1.5 m height and then a solid ice film formed. Afterward, the frozen ice film was annealed and last for 50 min to analyze the IRI activity of WSe_2_‐PVP NPs. The digital camera can capture the images of ice crystals in real time. Subsequently, a certain amount of largest ice grains in the field of view were chosen to elevate the average mean ice area using Image J processing software. All the samples were repeated at least three times and the error bar represented the standard deviation of size distribution of ice crystals. For the devitrification experiment of CPA solution with and without WSe_2_‐PVP NPs, the 5 µL solution was added onto the glass. Similar with previous report,^[^ ^73^ ^]^ the sample was cooled from 25 to −4 °C and waited for 2 min, then the sample was cooled at 50 °C min^−1^ and warmed under 20 °C min^−1^. The temperature when the vision suddenly darkened was recorded as devitrification temperature.

##### Rapid Rewarming by Photothermal Transformation Effect

A 808 nm laser (Laserver Inc., China) was used to irradiate the PS with vitrification solution with different concentrations of WSe_2_‐PVP NPs (0.1, 0.3, and 0.5 mg mL^−1^) under 3 W cm^−2^ laser power energy for 10 s. Compared with conventional water bath thawing, no visible ice formation were observed during rewarming process, thus indicating that the laser thawing suppressed the occurrence of devitrification and/or recrystallization and facilitated the cell recovery.

##### DSC Analysis Experiment

DSC‐204F1 instrument was used to assess the effect of PVP on the ice formation of CPA solution (1 m 1,2‐propanediol (PG), 1 m ethylene glycerol (EG), 1.2 m trehalose). The sample was cooled from 25 to −150 °C at 5 °C min^−1^ without ice seeding and held for 3 min to fully freeze sample. Then the sample was annealed to 25 °C at 10 °C min^−1^. Heat flow (mW mg^−1^) and melting enthalpy (J g^−1^) were recorded and used to evaluate the crystallization behaviors of the mixed solution. Moreover, for the devitrification analysis of CPA solution, in order to attain comparable cooling condition with rapid‐cooling method, the samples were directly immersed into liquid nitrogen during cooling procedure. Then the warming process was recorded in real time from −150 to 20 °C at 10 °C min^−1^.

##### Uptake of Tungsten Diselenide‐Polyvinyl Pyrrolidone Nanoparticles

HUVEC‐laden constructs were incubated with WSe2‐PVP NPs for 6 h at 4 ℃. After the HUVECs were released from the microcapsules, the cells were washed with PBS three times and fixed with glutaraldehyde at 4 ℃ overnight for the preparation of the biological samples for TEM analysis (TEM; Hitachi, Ltd., Tokyo, Japan).

##### Cell Culture

HUVECs were purchased from Hefei Node Biological Technology Co., Ltd. The cells were cultured with medium (high glucose) containing 10% fetal bovine serum and 1% penicillin/streptomycin at 37 °C in the incubator humidified 5% CO_2_ condition. The cell medium was changed every 2 days and the cells were detached for cell cryopreservation experiment until the cell density reached 80% approximately.

##### Cytotoxicity of Tungsten Diselenide‐Polyvinyl Pyrrolidone Nanoparticles

Cell Counting Kit‐8 (CCK‐8) was used to elevate the cell viability to analyze the toxicity of WSe_2_‐PVP NPs. The HUVECs were seeded in a 96‐well plate (BD Biosciences, NJ, USA) and cultured using the culture medium with 0.1, 0.3, and 0.5 mg mL^−1^ WSe_2_‐PVP NPs at 37 °C in a humidified incubator for 6 h. At the same time, the HUVECs were also cultured in the culture medium without WSe_2_‐PVP NPs as a control group. Afterward, 8 µL CCK‐8 reagent (Beyotime Institute of Biotechnology, China) was added per well and incubated for 4 h in a humidified incubator. Eventually absorbance was analyzed at 450 nm using a microplate reader (Diagnostics Pasteur, Marne la Coquette, France). The cell viability was measured according to the ratio of the absorbance to that of the control group at different conditions.

##### Fabrication of Human Umbilical Vein Endothelial Cell‐Laden Construct

An efficient and simple centrifugal microfluid was utilized to fabricate HUVEC‐laden constructs, which had been reported in our previous work.^[^ ^74^ ^]^ HUVECs were incorporated into sodium alginate solution (2%, w/v), once the alginate solution contacted with CaCl_2_ solution (1.5%, w/v) under centrifugal force and then the constructs formed. The optical images show the morphology of hydrogel microcapsules and insert picture exhibits the HUVEC‐laden construct. The size distribution of hydrogel microcapsule is from 250 to 325 µm and mean diameter is 273 µm (Figure S13b, Supporting Information).

##### Cryopreservation of Human Umbilical Vein Endothelial Cell‐Laden Constructs

The cryopreservation of HUVEC‐laden constructs was conducted according to the strategy that had been reported in the previous work.^[^ ^75^ ^]^ The CPA solution consisted of 1 m PG, 1 m EG, 1.2 m trehalose and with 0.1, 0.3, and 0.5 mg mL^−1^ WSe_2_‐PVP NPs in culture medium with 50% fetal bovine serum. As a controlled group, 10% DMSO (v/v) was added into culture medium with 50% (v/v) fetal bovine serum. The HUVEC‐laden constructs containing 1 m PG and 1 m EG were incubated at 4 °C for 20 min. Then the CPAs were removed and the samples were incubated into the CPAs solution with certain concentration of WSe_2_‐PVP NPs at 4 °C for 10 min. Ultimately, a certain volume of samples were transferred into a PS (FHK, Japan) and the PS was immersed into liquid nitrogen as soon as possible. After achieving complete vitrification and full thermal equilibrium, the samples were thawed by quickly plunging into 37 °C water bath with laser photothermal heating under 3 W cm^−2^ power density. The CPAs were stepwise removed by culture medium containing 0.75, 0.5, 0.25 m trehalose and pure culture medium, respectively.

##### Cell Viability Assays

The viability of cell post cryopreservation within 2 h was measured by using the acridine orange/ethidium bromide (AO/EB) Staining kit (KeyGen Biotech Co., Ltd., Nanjing, China). Furthermore, the long‐time cell viability assessment post cryopreservation was conducted after injecting in vivo at 1, 3, 5, 7 days, respectively. All the HUVEC‐laden construct samples cryopreservation experiments were repeated three times.

##### Human Umbilical Vein Endothelial Cell‐Laden Constructs Culture In Vivo and Viability Assessment

7‐week old female BLAB/c mice purchased from Vital River Laboratory Animal Technology Co., Ltd (Beijing, China) were used as experimental animal receptors to culture cryopreserved HUVEC‐laden cultures. All the animal studies got permission from the ethics committee of University of Science and Technology of China. Themice were anaesthetized and hair of back were shaved, and then the cryopreserved HUVEC‐laden constructs stored in culture medium were injected into hypodermis of mouse back, the wound of every mouse was sutured (Figure S16, Supporting Information). After cultured in vivo for 1, 3, 5, 7 days, the corresponding cryopreserved HUVEC‐laden constructs were taken out and the viabilities of constructs were evaluated using immunofluorescence technology. Furthermore, the cell constructs that had been cultured at seventh day were observed by laser scanning confocal microscopy for obtaining clearer results.

##### Statistical Analysis

All the experimental data are expressed as mean ± standard deviation from at least three independent experiments. Statistical analysis was calculated using a Student's two‐tailed unpaired *t*‐tests. (*p* < 0.05 was considered statistically significant)

## Conflict of Interest

The authors declare no conflict of interest.

## Supporting information

Supporting Information
