# Supplementary material for: Synergistic Ice Inhibition Effect Enhances Rapid Freezing Cryopreservation with Low Concentration of Cryoprotectants
Source: Adv Sci (Weinh). 2021 Jan 29;8(6):2003387. doi: 10.1002/advs.202003387 (PMC7967066; doi:10.1002/advs.202003387)
Supplement: Supplementary file 1 — Supporting Information [file ADVS-8-2003387-s001.pdf]

© 2021 Wiley-VCH GmbH

ADVANCED SCIENCE
Open Access

Supporting Information

for *Adv. Sci.,* DOI: 10.1002/advs.202003387

Synergistic Ice Inhibition Effect Enhances Rapid Freezing Cryopreservation with Low Concentration of Cryoprotectants

Tie Chang,[1#] *Oyawale Adetunji Moses,*[2#] *Conghui Tian,*[1] *Hai Wang,*[3,4*] *Li Song,*[2*] *Gang Zhao*[1,5*]

Supporting Information

Synergistic Ice Inhibition Effect Enhances Rapid Freezing Cryopreservation with Low Concentration of Cryoprotectants

Tie Chang,[1#] Oyawale Adetunji Moses,[2#] Conghui Tian,[1] Hai Wang,[3,4*] Li Song,[2*] Gang Zhao[1,5*]

[1] Department of Electronic Science and Technology, University of Science and Technology of China, No. 96 Road Jinzhai, Hefei 230027, Anhui, China

[2] National Synchrotron Radiation Laboratory, CAS Center for Excellence in Nanoscience, University of Science and Technology of China, Hefei 230029, Anhui, China

[3] CAS Key Laboratory for Biomedical Effects of Nanomaterials and Nanosafety, CAS Center for Excellence in Nanoscience, National Center for Nanoscience and Technology, Beijing 100190, China

[4] University of Chinese Academy of Sciences, Beijing 100049, China

[5] Anhui Provincial Engineering Research Center for Biopreservation and Artificial Organs, Anhui Medical University, Hefei 230022, Anhui, China

# Contributed equally

*Author to whom correspondence should be addressed.

Email:

zhaog@ustc.edu.cn (GZ), song2012@ustc.edu.cn (LS),wanghai@nanoctr.cn(HW).

Keyword: cryopreservation, ice inhibition, synergistic effect, tungsten diselenide-polyvinyl pyrrolidone nanoparticles

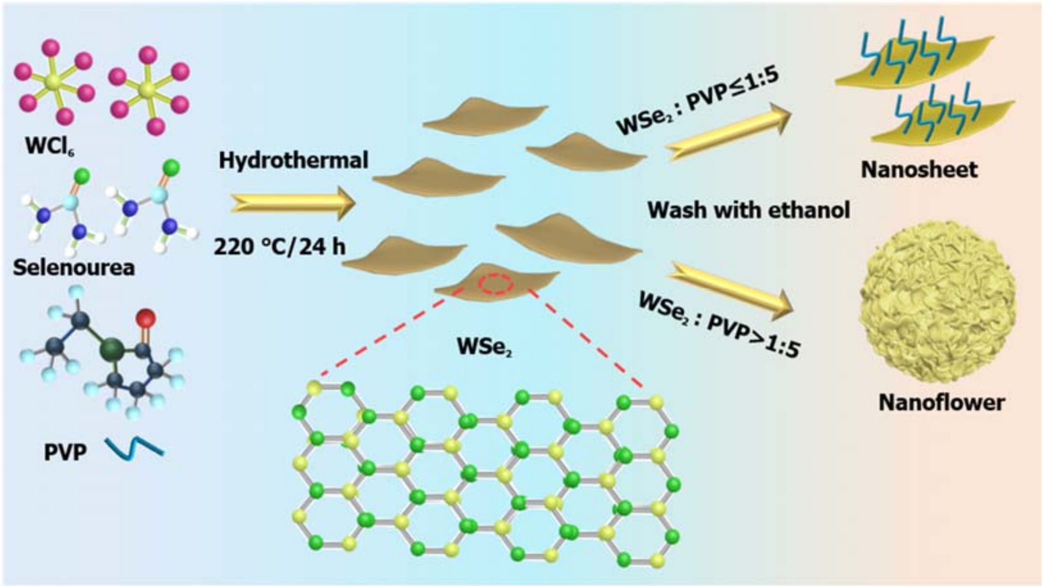

Figure S1: The synthesis process and schematic model of WSe_2-PVP nanosheets and nanoflowers.

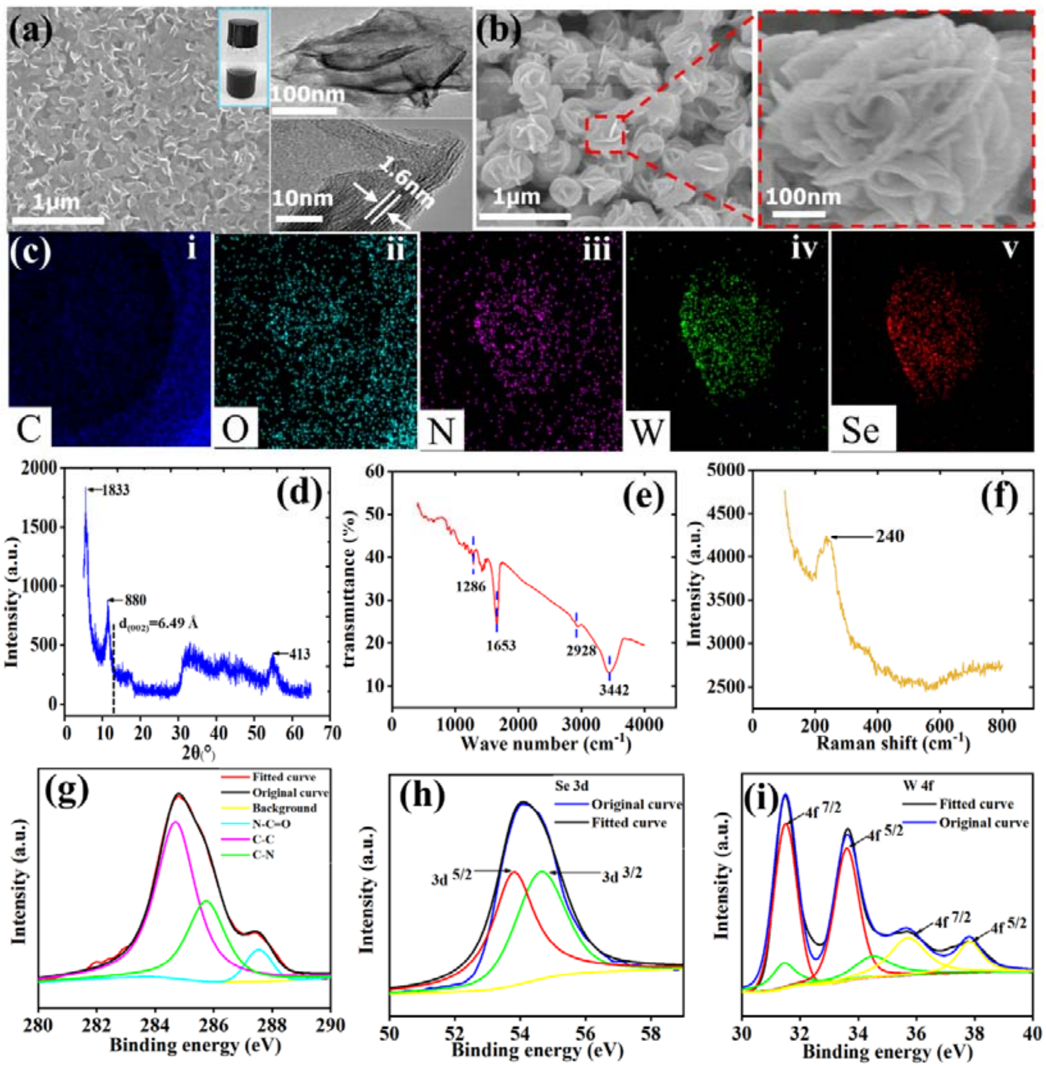

Figure S2: Materials characterization. (a) Typical SEM, TEM and HRTEM images of WSe_2-PVP NPs. The insert image is a typical photography of WSe_2-PVP NPs solution with well dispersibility. (b) SEM images of WSe_2-PVP nanoflowers. (c) EDS mapping images of C, O, N, W and Se. (d) XRD pattern of WSe_2-PVP NPs. (e) FT-IR spectra of WSe_2-PVP NPs. (f) Raman spectra of WSe_2-PVP NPs. (g-i) C 1s, W 4f and Se 3d XPS spectrum of WSe_2-PVP NPs.

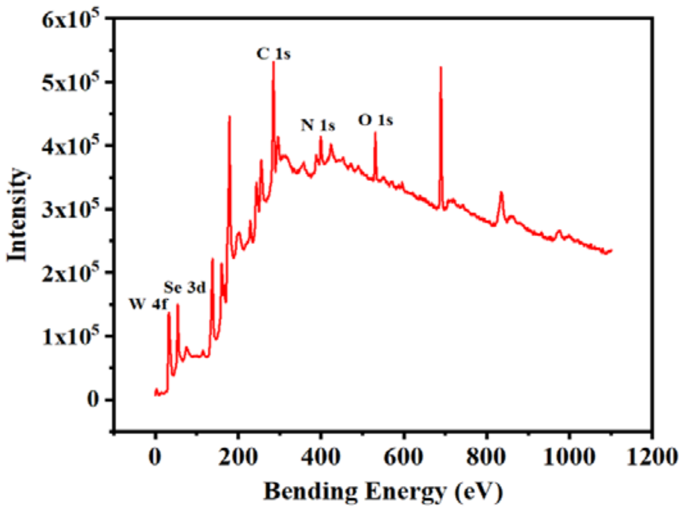

Figure S3: XPS survey spectra of the obtained WSe_2-PVP NPs.

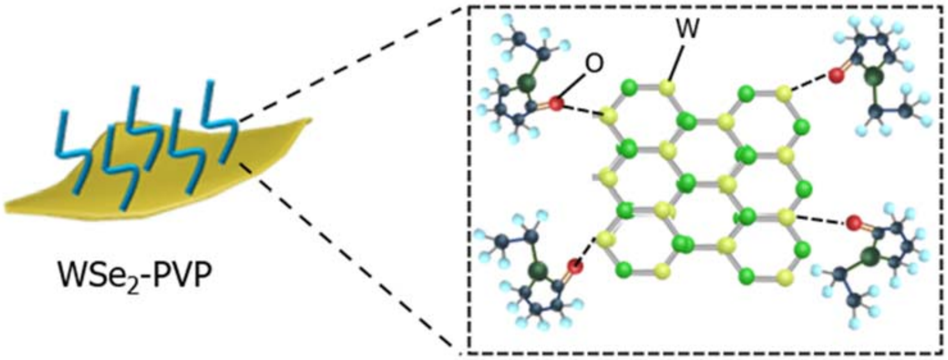

Figure S4: The proposed chemical reaction mechanism between PVP and WSe_2 based on chelating-coordinating effect between the lone-pair electrons of PVP carbonyl oxygen and the unoccupied 5d orbitals of W.

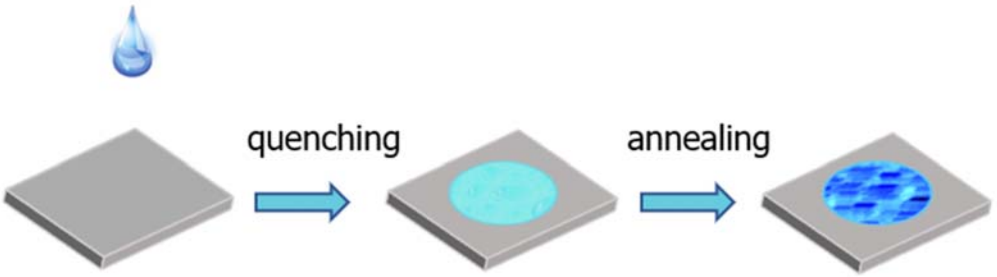

Figure S5: Schematic illustration of “splat cooling” experiment.

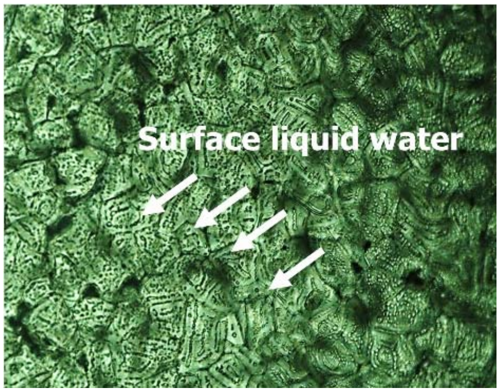

Figure S6: Surface liquid water occurred onto the ice surface accelerates the growth of ice grain.

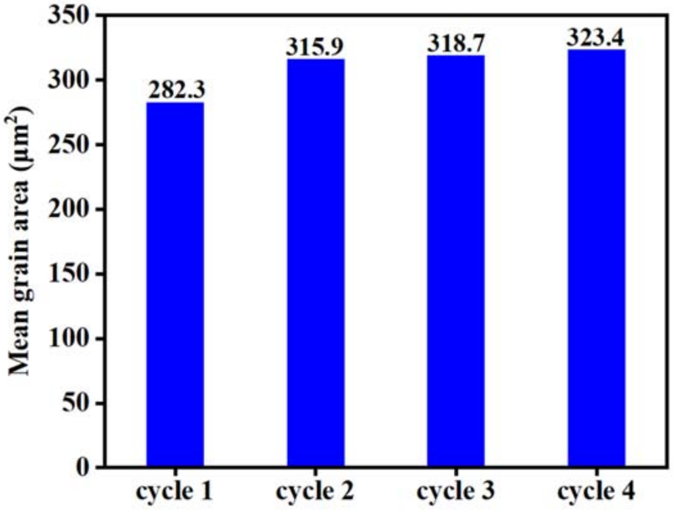

Figure S7: Mean areas of ice crystals after four quenching and annealing cycles. After undergoing four cycles, the mean areas of ice crystals are similar, indicating the IRI activity of WSe_2-PVP NPs of is stable.

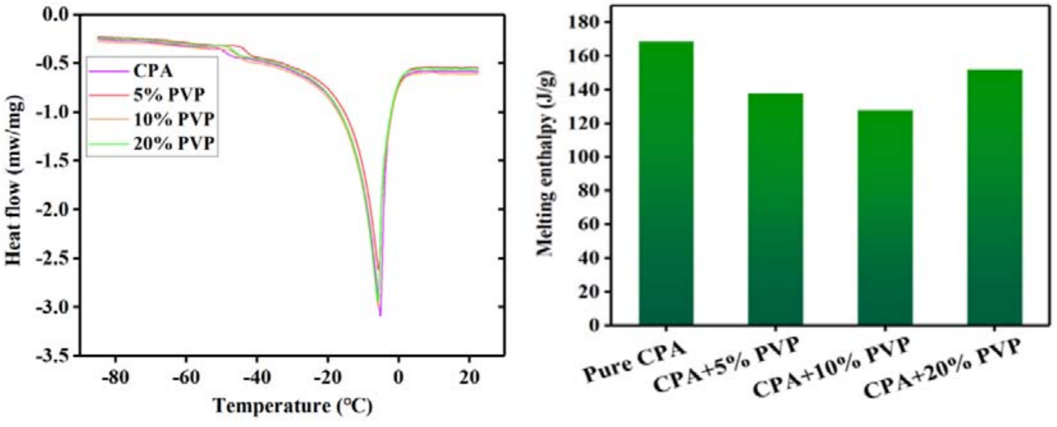

Figure S8: Heating DSC curves for CPA solution with different concentrations of PVP and corresponding melting enthalpy.

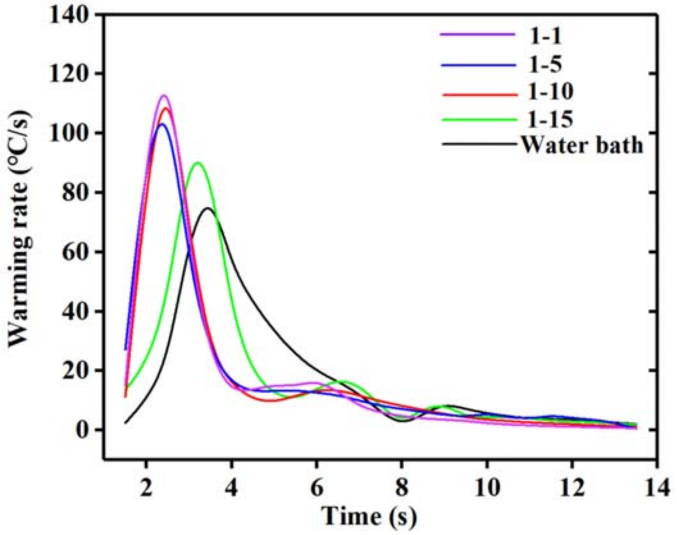

Figure S9: The comparison of rewarming rates of vitrification solution with four types of WSe_2-PVP NPs under laser irradiation from nitrogen to water bath.

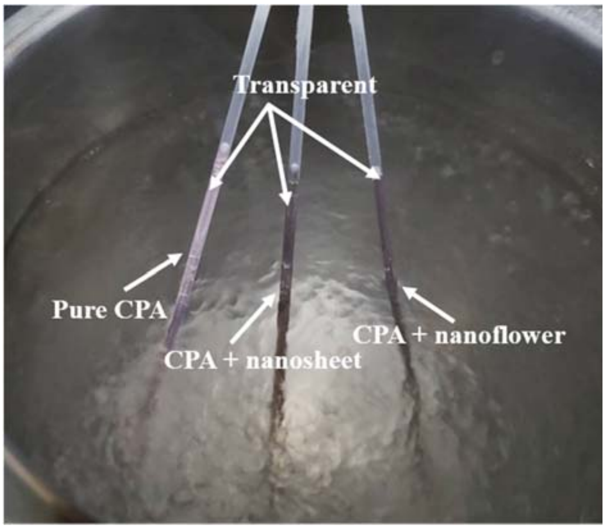

Figure S10: Transparent appearance of CPA solution with and without nanoparticles during cooling process.

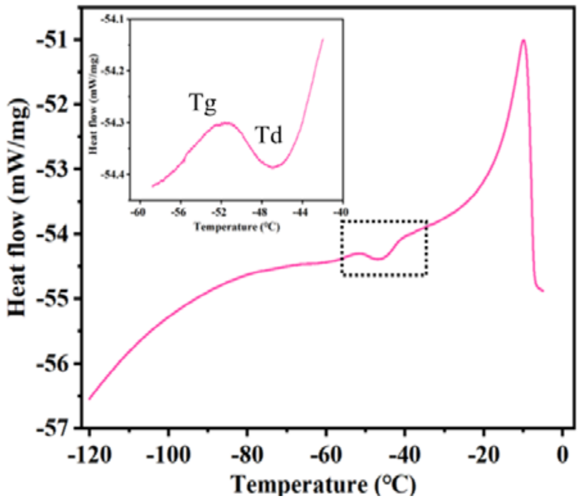

Figure S11: The occurrence of devitrification for CPA solution by DSC analysis.

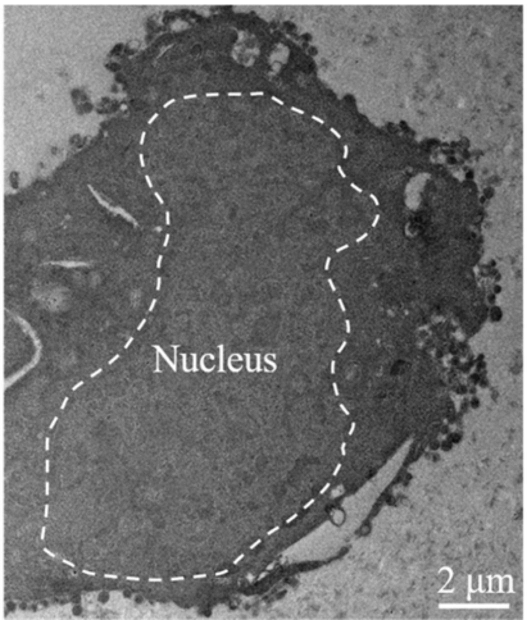

Figure S12: TEM image of HUVEC-laden constructs after incubation with WSe_2-PVP NPs for 6 h.

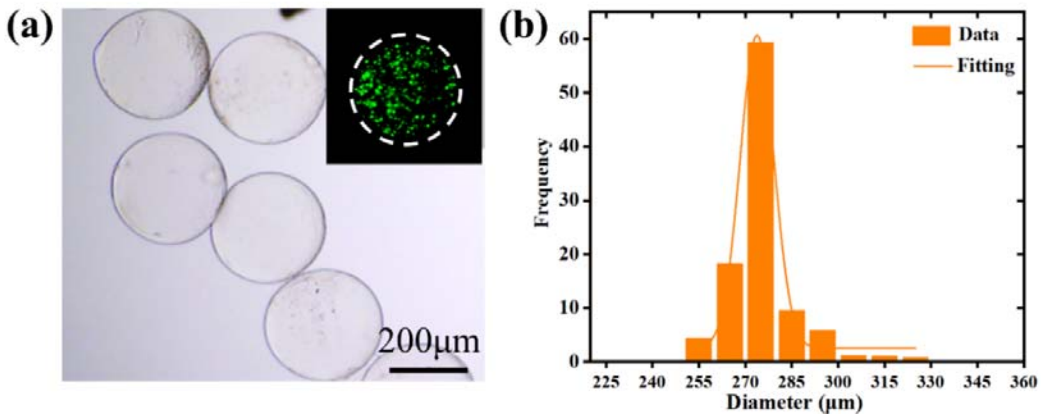

Figure S13: (a) Optical images of monodispersed hydrogel microcapsules built using centrifugal microfluid. Insert image shows the HUVEC-laden hydrogel microcapsule after fluorescence. (b) Size distribution of hydrogel microcapsules.

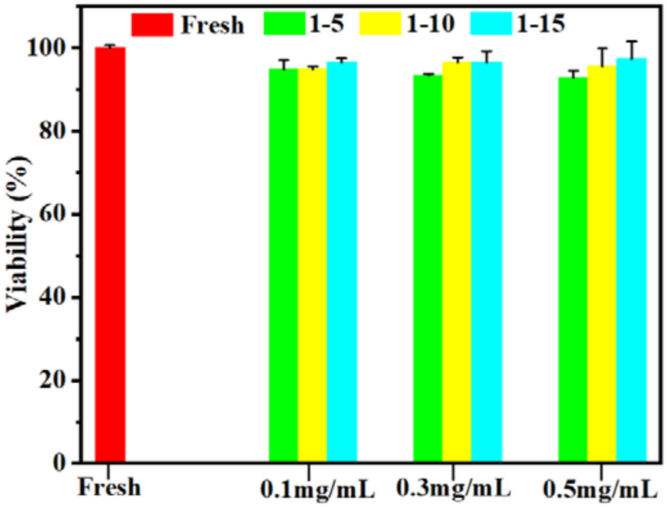

Figure S14: Cytotoxicity of HUVECs at three types of WSe_2-PVP NPs under different concentrations determined by viability for 6 h at 37 °C.

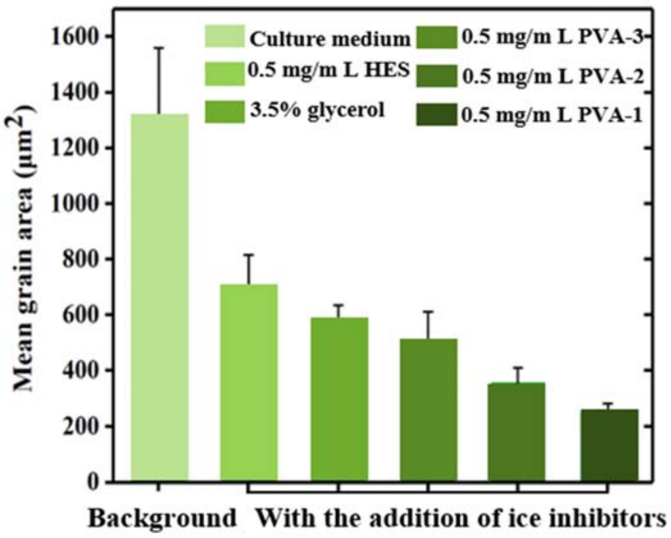

Figure S15: Quantitative assessment of grain area of ice crystals obtained from culture medium with addition of HES, glycerol, and PVA for comparison.

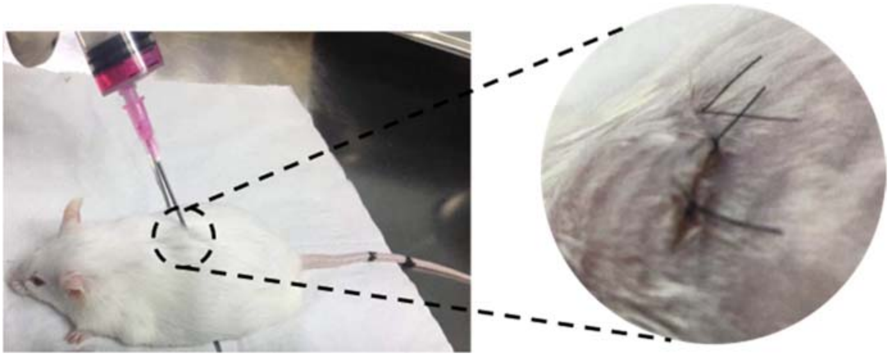

Figure S16: Images of hypodermis injection and wound closure on the back of mouse.
